# RegiSTORM: channel registration for multi-color stochastic optical reconstruction microscopy

**DOI:** 10.1186/s12859-023-05320-1

**Published:** 2023-06-05

**Authors:** Øystein Øvrebø, Miina Ojansivu, Kimmo Kartasalo, Hanna M. G. Barriga, Petter Ranefall, Margaret N. Holme, Molly M. Stevens

**Affiliations:** 1 Department of Materials, Imperial College London, London, SW7 2AZ UK; 2 Department of Bioengineering, Imperial College London, London, SW7 2AZ UK; 3 Institute of Biomedical Engineering, Imperial College London, London, SW7 2AZ UK; 4 Department of Medical Biochemistry and Biophysics, Karolinska Institute, 171 77 Stockholm, Sweden; 5 Department of Medical Epidemiology and Biostatistics, Karolinska Institute, 171 77 Stockholm, Sweden; 6 SciLifeLab BioImage Informatics Facility, and Department of Information Technology, Uppsala University, 751 05 Uppsala, Sweden

**Keywords:** Super-resolution microscopy, Stochastic optical reconstruction microscopy, Single-molecule localization microscopy, Registration, Multi-channel, Image analysis tool, Software

## Abstract

**Background:**

Stochastic optical reconstruction microscopy (STORM), a super-resolution microscopy technique based on single-molecule localizations, has become popular to characterize sub-diffraction limit targets. However, due to lengthy image acquisition, STORM recordings are prone to sample drift. Existing cross-correlation or fiducial marker-based algorithms allow correcting the drift within each channel, but misalignment between channels remains due to interchannel drift accumulating during sequential channel acquisition. This is a major drawback in multi-color STORM, a technique of utmost importance for the characterization of various biological interactions.

**Results:**

We developed RegiSTORM, a software for reducing channel misalignment by accurately registering STORM channels utilizing fiducial markers in the sample. RegiSTORM identifies fiducials from the STORM localization data based on their non-blinking nature and uses them as landmarks for channel registration. We first demonstrated accurate registration on recordings of fiducials only, as evidenced by significantly reduced target registration error with all the tested channel combinations. Next, we validated the performance in a more practically relevant setup on cells multi-stained for tubulin. Finally, we showed that RegiSTORM successfully registers two-color STORM recordings of cargo-loaded lipid nanoparticles without fiducials, demonstrating the broader applicability of this software.

**Conclusions:**

The developed RegiSTORM software was demonstrated to be able to accurately register multiple STORM channels and is freely available as open-source (MIT license) at https://github.com/oystein676/RegiSTORM.git and 10.5281/zenodo.5509861 (archived), and runs as a standalone executable (Windows) or via Python (Mac OS, Linux).

**Supplementary Information:**

The online version contains supplementary material available at 10.1186/s12859-023-05320-1.

## Background

Fluorescence microscopy is a widely utilized tool in cell and molecular biology, providing detailed structural information of specifically labeled target structures/molecules and their interactions. However, with conventional microscopic techniques the diffraction limit of light constrains the achievable resolution to roughly 200 nm. This major limitation has led to the development of super-resolution imaging methods such as Stochastic Optical Reconstruction Microscopy (STORM) [1, 2]. In STORM the blinking of sample fluorophores is photophysically induced and chemically supported, followed by image recording over thousands of frames where each frame has a distinct subset of blinking events. The exact x,y coordinates of the blinks are then computationally determined at nanoscale resolution. Following this reconstruction process, pre-processed STORM data consists of sets of such datapoints indicating the locations of individual fluorophores. In the remainder of the paper, we refer to these datapoints as localizations. STORM data in the form of localizations can then be further analyzed as such, retaining the full resolution of the identified coordinates, or be used to generate super-resolved images.

Instead of visualizing only one target with fluorescence microscopy there is often a great interest in studying the interactions of multiple targets, necessitating multiple labels and multicolor imaging approaches. In diffraction-limited microscopy, this is straightforward as the image acquisition is quick, allowing multiple channels to be simply overlaid with no major (observable) error. In STORM, however, where the resolution is an order of magnitude higher than in diffraction-limited microscopy, various types of errors stemming from either the physics of light (e.g. chromatic aberration), image acquisition or image processing, are more frequently detected and need to be accounted for. Chromatic aberration and other imperfections in the optical imaging system are constant for a specific microscope setup and there are good tools available to easily correct these [3, 4]. Due to the lengthy image acquisition times in STORM (typically tens of minutes), sample drift is the main error source affecting image quality [5]. Drift is typically caused by mechanical movements in the hardware (e.g. sample stage, filter cube etc.) as well as temperature changes, which are stochastic and make the prediction or modeling of drift impossible. Within one channel the error caused by drift can be readily corrected using existing algorithms, which are often based on either fiducial markers or cross-correlation [5, 6]. However, in multi-color STORM imaging, especially when the channels are acquired in a sequential manner and there is a gap between the acquisitions due to filter change etc., tracking the drift becomes a problem.

To enable multi-color imaging without sequential channel acquisition, different approaches have been presented. In a spectral demixing approach a dichroic-based emission splitter is used to separate simultaneously and with the same wavelength excited fluorophores based on their different emission spectra [7–9]. Recently, a method called excitation-resolved STORM (ExR-STORM) was demonstrated to enable multi-color imaging with negligible chromatic aberration by separating signals from dyes with the same emission wavelength but different excitation wavelengths based on differences in fluorescence intensities [10]. However, all these methods require complex and expensive custom-built microscope hardware setups, making them not easily accessible for most of the users. Moreover, unlike in sequential STORM where the fluorescent probes are spectrally well-separated, simultaneous acquisition of multiple channels often suffers from spectral crosstalk.

Despite being easily executable with minimal spectral crosstalk, sequential channel acquisition has the inherent challenge of proper channel registration, or alignment, which is highly crucial for the error-free evaluation of nanoscale interactions (e.g. protein–protein, protein-organelle etc.). In some cases it is possible to do the channel alignment manually based on the known biological interactions of the imaged targets (e.g. ring-center) [11]. However, in most of the cases the interactions are not known. In such cases fiducial markers, non-blinking fluorescent nanobeads visible in all the channels, can be introduced to the sample to serve as reference points for the channel registration [12, 13]. To minimize the fiducial marker interference with the sample signal, fiducial markers excitable with an infrared light emitting diode can be used, though this system requires adjustments in the hardware [14]. In general, the fiducial marker based STORM channel registration is mostly done manually, making it labor-intensive and error-prone. Of note, there are also conventional general purpose image registration algorithms available, such as ImageJ/Fiji and Elastix [15–18], but they are poorly suited for STORM, since they operate on images, that is, arrays of pixel values. In the case of STORM, it is preferable to perform analysis directly on localization data. This retains the full spatial resolution and avoids the unnecessary step of first synthesizing super-resolved images. Moreover, identifying fiducial markers from super-resolved images using image processing approaches can be challenging if their morphology resembles that of the targets of interest, whereas identifying fiducials from localization data can be performed based on their lack of blinking.

Over the recent years multiple single-molecule localization microscopy (SMLM) data processing softwares, some of which are open-access and available for e.g. ImageJ or Matlab platforms, have been developed and thoroughly evaluated and cross-compared [19, 20]. Significant improvements have been achieved in e.g. localization accuracy, reconstruction speed and user-friendliness, and some of the softwares provide a real-time reconstruction pipeline to better enable image quality evaluation already during the frame acquisition [21–27]. Even deep-learning with neural networks has been utilized for SMLM, drastically reducing the amount of image data needed to obtain high-quality reconstructions [28]. In addition to reconstruction of the SMLM images, softwares have also been developed for automated detection, classification and quantification of the various (cellular) structures from reconstructed SMLM images [29–31]. However, to the best of our knowledge, none of the available open-source SMLM softwares are able to identify the fiducial markers from the localization data and perform channel registration using the fiducials as reference points.

In this work we have developed an algorithm, which can identify fiducial markers in STORM data, and use them to accurately register multiple sequentially acquired channels. Importantly, the algorithm operates directly on localization data, and also outputs the corrected data in the form of localizations. Of note, chromatic aberration was corrected separately prior to registration using the Detection of Molecules (DoM) plugin in Fiji [4], and therefore does not account for any of the drift seen in the data. This algorithm has been implemented in RegiSTORM, a Windows application featuring a user-friendly graphical user interface (GUI). Multicolor STORM image registration using RegiSTORM has been successfully applied to samples consisting of fiducial markers only and cell samples of multi-labeled tubulin with fiducial markers introduced prior to imaging. Moreover, we have included an additional fiducial marker independent function, called cluster mode, to the RegiSTORM software and illustrated its ability to succesfully register two-color STORM images of cargo-loaded lipid nanoparticles (LNPs). This further demonstrates the applicability of RegiSTORM in versatile cases.

## Results and discussion

**Fig. 1:**
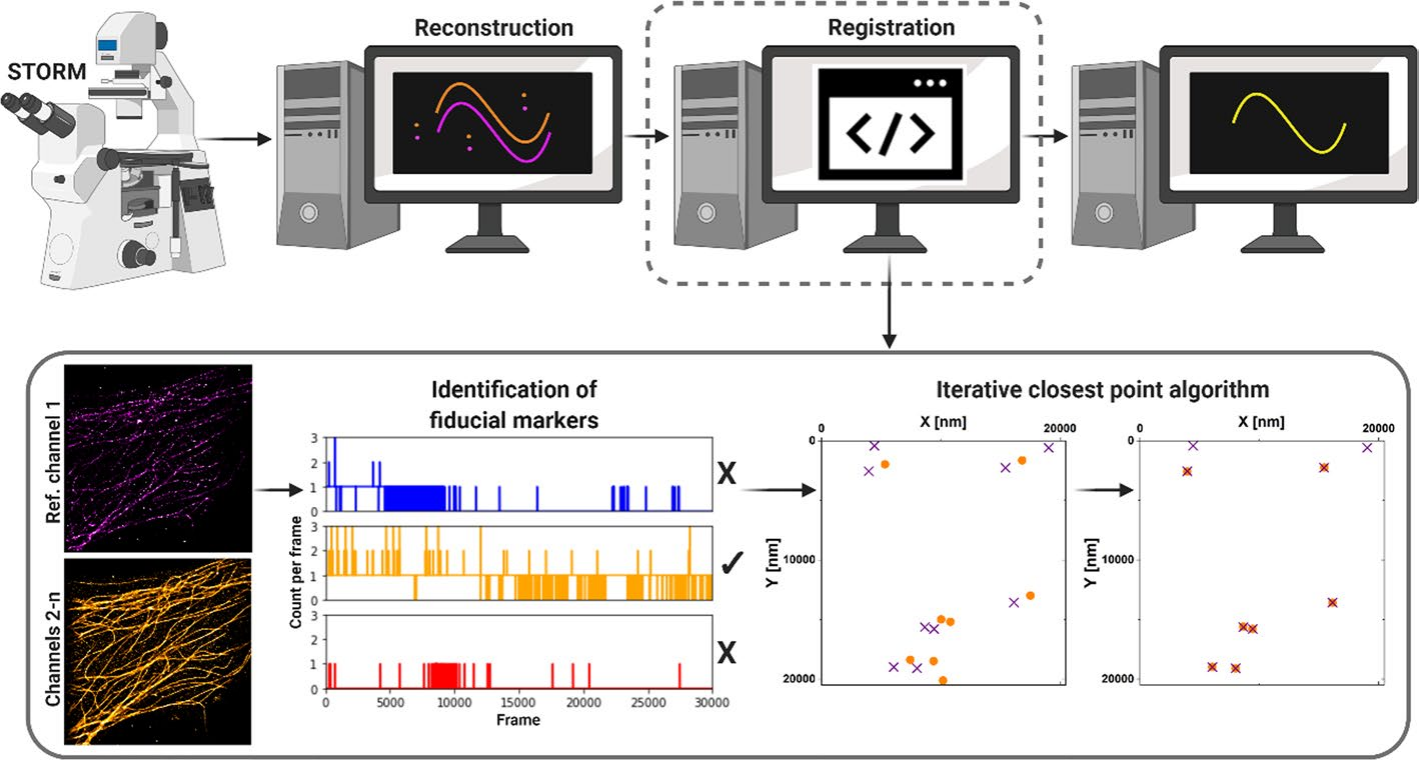
Schematic illustration of the registration algorithm. Top, from left: The algorithm takes the reconstructed STORM data (in the form of fluorophore x, y localizations) and applies a rigid transformation to the fluorophore localizations to perform registration between channels, producing localizations corrected for drift. Bottom, from left: Initially, fiducial markers are identified from each channel using two detection criteria: (1) The mean number of localizations present within a local neighborhood over time exceeds a given threshold, and (2) The variance of the number of localizations within the neighborhood over time is below a given threshold. As a result, non-blinking constantly emitting sources corresponding to the fiducial markers are retained. The rigid transformation between the channels is then estimated to align the fiducial markers, using the Iterative Closest Point algorithm [32–34]. The ICP algorithm is used to align two-point sets by minimizing the distance between corresponding points. It iteratively updates the transformation between the two sets until convergence using point-to-point correspondences and estimates a rigid transformation combining rotation and translation through a root mean square distance minimization

In this work we have developed an algorithm that utilizes fiducial markers to register multiple channels in STORM imaging to compensate for drift between the channel acquisitions. We have demonstrated the performance of the algorithm with several samples, including fiducial markers alone, multi-labeled tubulin in fixed cells and, using the cluster mode of the algorithm, cargo-loaded LNPs with no fiducials. These results are discussed in detail below. A schematic illustration of the RegiSTORM algorithm as part of the STORM imaging process is depicted in Fig. 1.

### Registration of 2- and 3-color STORM images of fiducial markers only

**Fig. 2:**
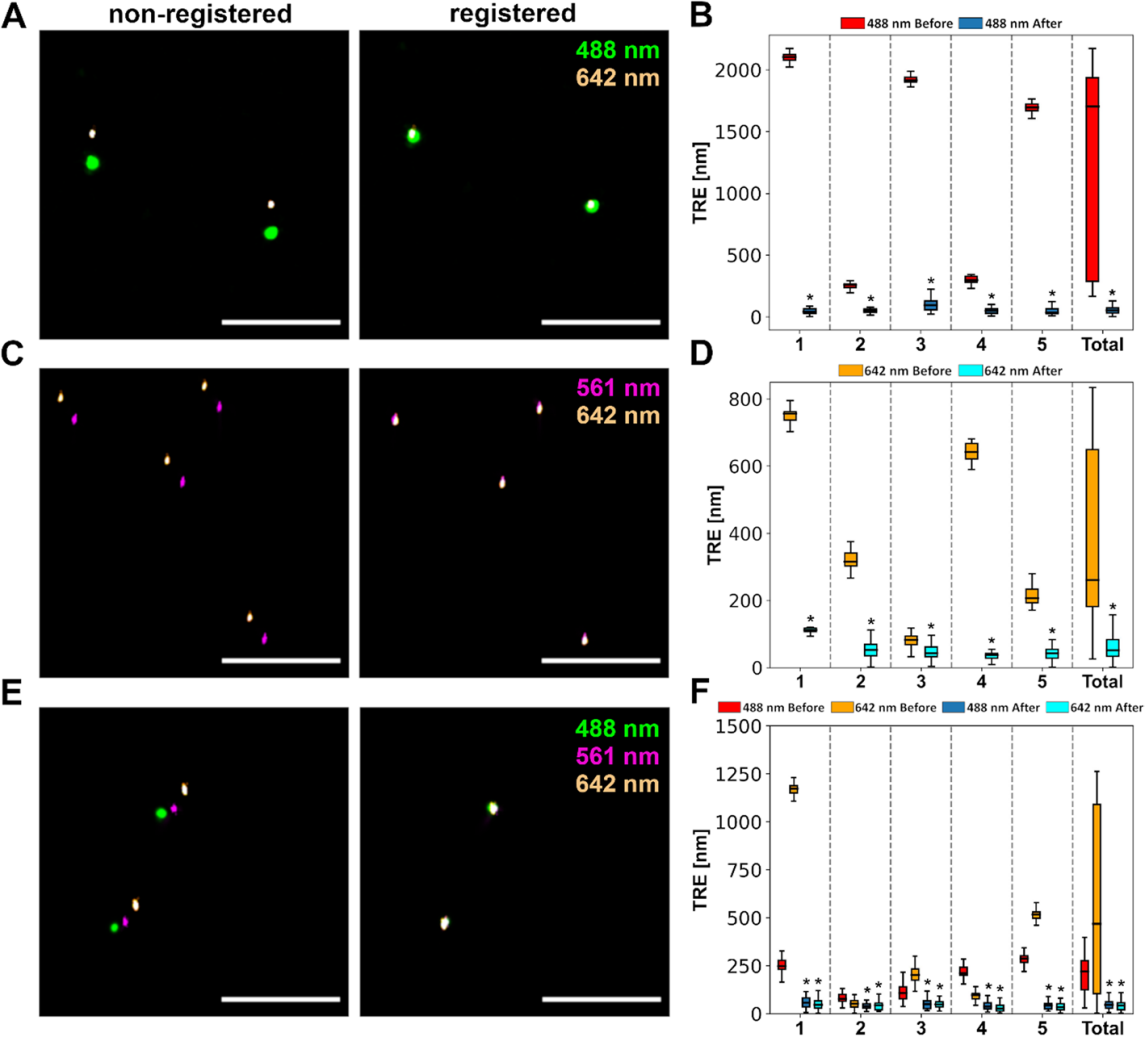
Evaluation of the registration algorithm with multi-color STORM images of fiducial markers only. **A** Representative STORM images of fiducial markers before and after registration, imaged with 488 and 642 nm excitation. **B** Quantification of the Target Registration Error (TRE) for A. 1–5 represent different 256 × 256 pixel areas (images) of the same sample. All the fiducial markers detected in the five different areas are combined in total. In the graph the different areas are separated by dashed lines and the «before» box is always on the left and «after» on the right. n1 = 26, n2 = 30, n3 = 40, n4 = 33, n5 = 29, n_total_ = 158. **C** Representative STORM images of fiducial markers before and after registration, imaged with 561 and 642 nm excitation. **D** Quantification of the TRE for C. n1 = 46, n2 = 37, n3 = 49, n4 = 22, n5 = 60, n_total_ = 214. **E** Representative STORM images of fiducial markers before and after registration, imaged with 488, 561 and 642 nm excitation. **F** Quantification of the TRE for E. n1 = 92, n2 = 48, n3 = 67, n4 = 57, n5 = 88, n_total_ = 352. Two-sided Wilcoxon signed rank test *p < 0.05. Scale bars in A, C and E = 1 μm. In the box plots the box shows the data from lower to upper quartile, with median marked with a line, whereas the whiskers indicate the minimum and maximum

To evaluate the performance of the algorithm we started from the simplest case, i.e. multi-color STORM images of fiducial markers alone, in the absence of any other signal which could complicate the detection of the fiducial markers. Using TetraSpeck™ fiducial markers we evaluated the following channel combinations: (1) 642 and 488 nm, (2) 642 and 561 nm and (3) 642, 561 and 488 nm. As the reference channels, we used 642 nm, 561 nm and 561 nm, respectively. Figure 2 shows representative STORM images of the fiducials imaged with these channel combinations as well as TRE plots, generated from localizations before and after the use of the algorithm. It is visually evident from the STORM visualizations that the algorithm markedly improves the overlay of the fiducial markers on the different channels. The TRE quantification, conducted for five separate samples, verified this observation. When combining the five samples (“total” in Fig. 2), there was a significant improvement (mean ± SD) from 1252 ± 806 nm to 60 ± 40 nm (n = 158, *p* = 1.1 × 10^–27^) for combination 1, from 362 ± 256 nm to 60 ± 36 nm (n = 214, *p* = 4.0 × 10^–36^) for combination 2, and from 496 ± 434 nm to 43 ± 24 nm & from 204 ± 86 nm to 49 ± 32 nm (n = 352, *p* = 4.5 × 10^–57^ & *p* = 1.4 × 10^–57^) for combination 3. These results demonstrate that the algorithm is capable of identifying the fiducials in the absence of other sample signals and successfully apply a rigid transformation to correct for the misalignment.

### Registration of 2- and 3-color STORM images of immunolabeled tubulin in cells

**Fig. 3:**
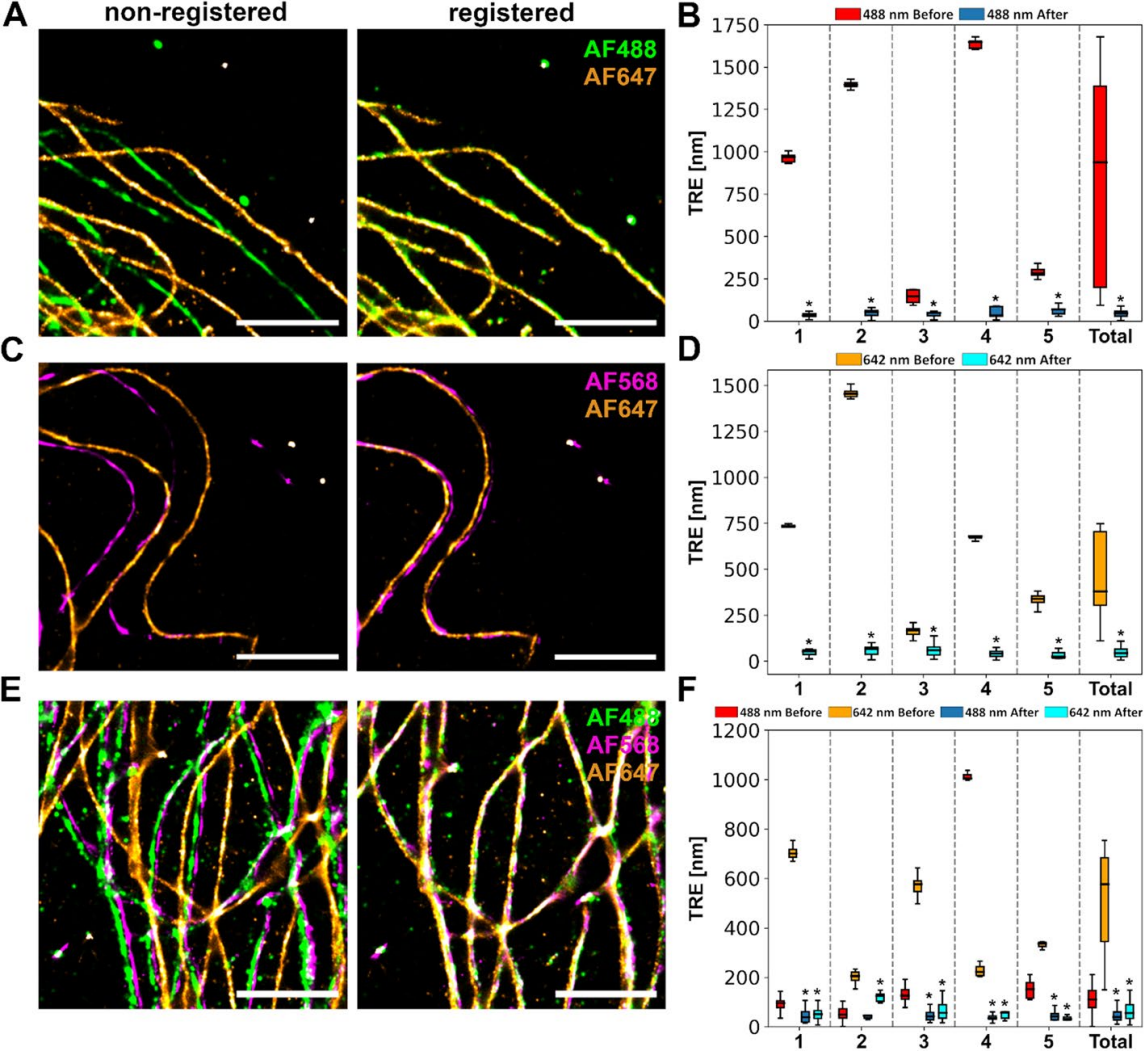
Evaluation of the registration algorithm with STORM images of cell samples with multi-labeled tubulin and fiducials. **A** Representative STORM images of dual-labeled tubulin before and after registration, imaged with 488 and 642 nm excitation. **B** Quantification of the Target Registration Error (TRE) of fiducial markers present in the samples in A. n1 = 11, n2 = 12, n3 = 13, n4 = 5, n5 = 10, n_total_ = 51. **C** Representative STORM images of dual-labeled tubulin before and after registration, imaged with 561 and 642 nm excitation. **D** Quantification of the TRE of fiducial markers present in the samples in C. n1 = 5, n2 = 11, n3 = 15, n4 = 15, n5 = 19, n_total_ = 65. **E** Representative STORM images of triple-labeled tubulin before and after registration, imaged with 488, 561 and 642 nm excitation. **F** Quantification of the TRE of fiducial markers present in the samples in E. n1 = 27, n2 = 10, n3 = 35, n4 = 6, n5 = 6, n_total_ = 84. Two-sided Wilcoxon signed rank test *p < 0.05. 1–5 represent different 256 × 256 pixel areas (images) of the same sample. All the fiducial markers detected in the five different areas are combined in total. In the graphs the different areas are separated by dashed lines and the «before» box is always on the left and «after» on the right. AF = Alexa Fluor. Scale bars in A, C and E = 2 μm. In the box plots the box shows the data from lower to upper quartile, with median marked with a line, whereas the whiskers indicate the minimum and maximum

To demonstrate the algorithm’s functionality in a more complex and biologically relevant setup, microtubules of human bone marrow-derived stromal cells (hBMSCs) were multi-labeled via immunostaining with a mixture of fluorophore-tagged secondary antibodies. Therefore, in case of successful registration, the microtubules in the different channels will overlap in the final reconstruction, making it possible to evaluate the performance of the algorithm under more realistic conditions. Importantly, the registration was still only based on the fiducials and not aided by the microtubule structures appearing in both channels. The same channel combinations ((1) 642 and 488 nm, (2) 642 and 561 nm, (3) 642, 561 and 488 nm) and reference channels (642 nm, 561 nm and 561 nm) were used as for the samples with fiducial markers only. Figure 3 shows representative tubulin STORM images imaged with these channel combinations as well as TRE plots, before and after the registration, calculated for manually annotated fiducial markers present in the samples. The STORM visualizations show a clear improvement in the microtubule overlay after the registration for all the channel combinations. This improvement was also quantified using the TRE for five different samples. Considering all five samples together, there was a significant improvement (mean ± SD) from 790 ± 565 nm to 49 ± 27 nm (n = 51, *p* = 5.2 × 10^–10^) for combination 1, from 593 ± 436 nm to 49 ± 32 nm (n = 65, *p* = 2.6 × 10^–12^) for combination 2, and from 177 ± 239 nm to 47 ± 46 nm & from 527 ± 181 nm to 65 ± 47 nm (n = 84, *p* = 4.7 × 10^–14^ & *p* = 1.8 × 10^–15^) for combination 3. The quality of the registration was further evaluated with Normalized Cross-Correlation (NCC), which also showed a clear overall improvement in the overlay as indicated by the increase in correlation coefficient values (Additional file 1: Table S1). Normalized Cross-Correlation (NCC) is a technique used in image registration that measures the similarity between two images by computing the correlation between their pixel intensities after normalization [35]. Due to competing secondary antibodies (i.e. steric hindrance on perfect nanoscale labeling) [36] and differing behavior of fluorophores under STORM acquisition, the absolute values of the coefficients in the NCC analysis remained relatively low even after successful registration. Overall, these results show that the algorithm is capable of identifying the fiducials and successfully applying a rigid transformation to correct for the channel misalignment also in the presence of sample fluorescence, demonstrating the feasibility of RegiSTORM in an experimentally relevant case.

### Registration of 2-color STORM images of cargo-loaded LNPs using the cluster mode

**Fig. 4:**
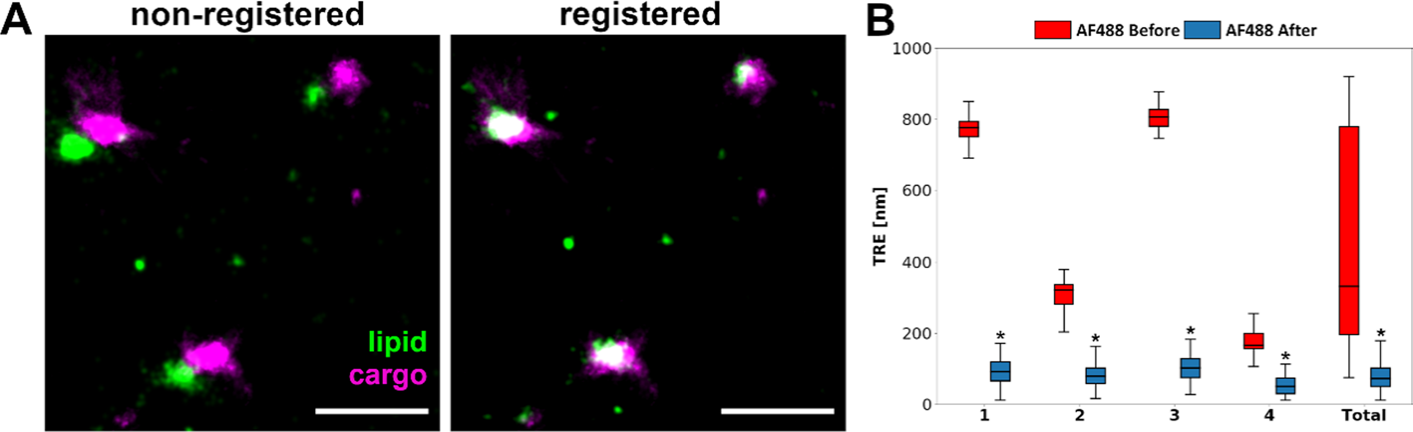
Evaluation of the cluster mode of the algorithm with two-color STORM images of cargo-loaded LNPs. **A** Representative STORM images of cargo-loaded LNPs before and after registration, imaged with 488 and 642 nm excitation. Scale bars = 1 μm. **B** Quantification of the Target Registration Error (TRE) of the LNP/cargo particles. n1 = 38, n2 = 29, n3 = 24, n4 = 50, n_total_ = 141. Two-sided Wilcoxon signed rank test *p < 0.05. 1–4 represent different 256 × 256 pixel areas (images) of the same sample. All the LNP/cargo particles detected in the four different areas are combined in total. In the box plots the box shows the data from lower to upper quartile, with median marked with a line, whereas the whiskers indicate the minimum and maximum

In addition to the fiducial marker based STORM channel registration described above, an alternative STORM image registration mode based on detection of clusters of localization signals originating from targets labeled by multiple fluorophores instead of fiducial markers was developed and tested. This mode is ideal for small targets such as various types of (multi-labeled) nanoparticles which, depending on the labeling approach, may produce cluster-like signal signatures in STORM. We hypothesized that these signal clusters could be used as landmarks for registration in the absence of fiducial markers. Omitting fiducial markers from these types of samples can be beneficial for the STORM acquisition since matching the signal intensity from the relatively bright fiducial markers to that of structures within the samples, which often are much dimmer, can be challenging and easily hampers the image quality. To demonstrate the functionality of the cluster mode we imaged streptavidin-Alexa Fluor 647 loaded LNPs labeled with Alexa Fluor 488 lipid (Fig. 4). As evidenced by the STORM visualizations of Fig. 4A the cluster mode of the algorithm markedly improved the channel alignment. This result was also quantified by manually annotating the clusters using the ImageJ annotation tool (see Methods) and calculating the mean TRE, demonstrating a total improvement (mean ± SD) from 474 ± 285 nm to 79 ± 43 nm (n = 141, *p* = 7.4 × 10^–25^) (Fig. 4B). Moreover, there was also an improvement in the NCC with the mean values of correlation coefficients increasing from 0.12 ± 0.14 to 0.50 ± 0.12 (Additional file 1: Table S2). The successful registration using the cluster mode demonstrates the versatility of the tool and broadens its applicability in the field of multicolor STORM imaging.

### Parameter tuning

**Fig. 5:**
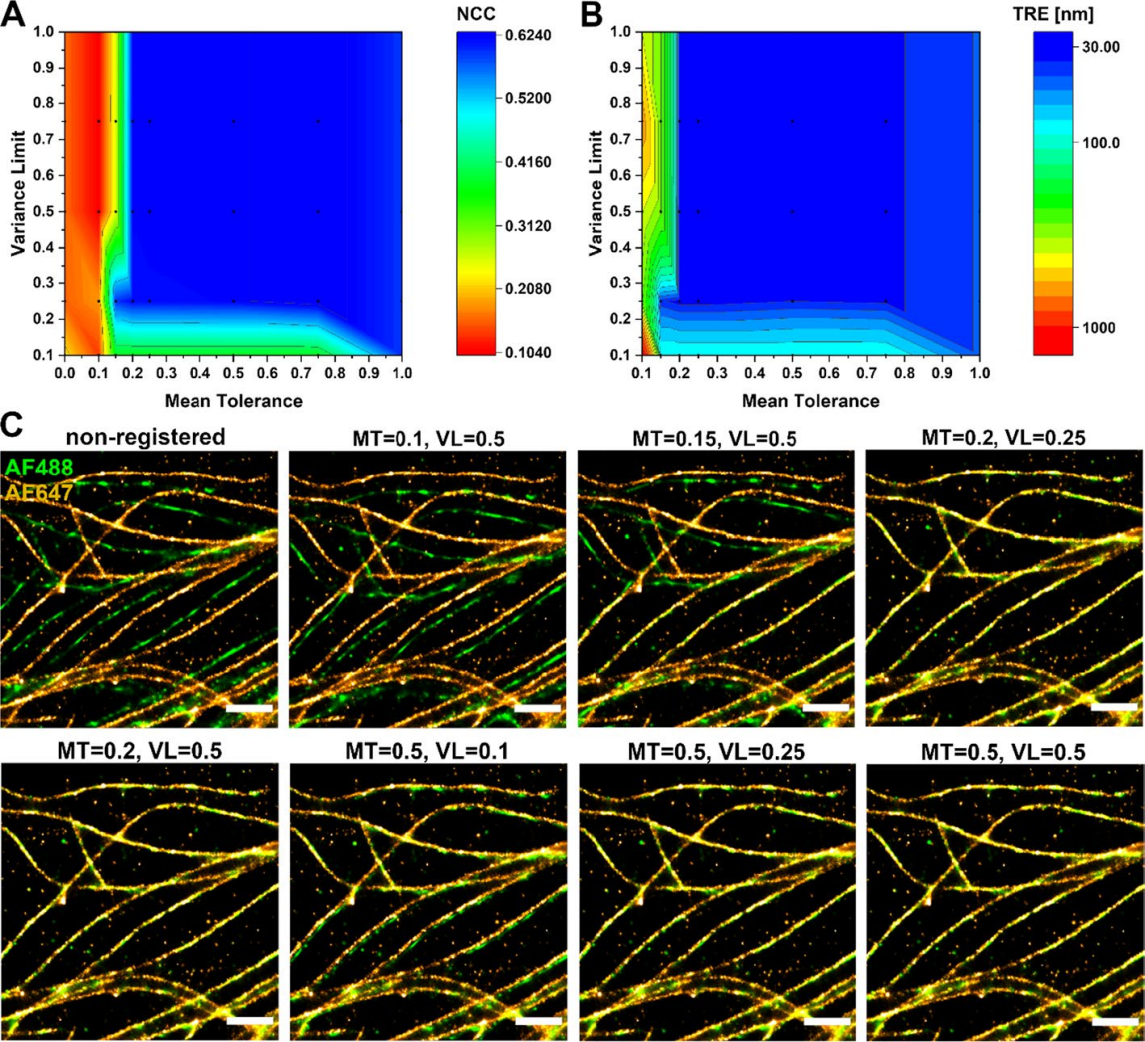
The effect of the registration parameters on the registration accuracy. **A** Normalized Cross-Correlation (NCC) as a function of variance limit and mean tolerance. **B** Target Registration Error (TRE) as a function of variance limit and mean tolerance. **C** Representative STORM images of dual-stained tubulin samples, registered with varying parameters. Scale bars = 1 μm. MT = mean tolerance, VL = variance limit, AF = Alexa Fluor

The algorithm uses two main tunable parameters when detecting the fiducials: mean tolerance (MT) and variance limit (VL). The MT helps to identify fiducial markers based on their stable emission across frames, while excluding isolated signals. However, when testing the algorithm on tubulin samples, we observed that the algorithm struggled to differentiate the fiducials from the microtubuli that also gave out frequent signals. Therefore, we introduced the VL, which defines a maximum allowed value for the variance of the number of nearby localizations across frames for a fiducial candidate. In the tubulin regions of the images we observed excessive blinking in the fluorescence signal, as expected, resulting in a high variance, whereas the fiducials are characterized by a constant stable signal and thus had a low variance. Therefore, by combining the MT, which finds relevant regions, and the VL, which distinguishes fiducials from densely labeled sample regions, it is possible to identify the fiducial marker localizations from the STORM data. The effect of adjusting MT and VL on the registration quality of two-color tubulin STORM images, as determined by TRE and NCC, is depicted as heat maps in Fig. 5A and B, respectively. Figure 5C shows representative STORM images of parameter-influence on the registration for selected MT/VL combinations. We found that an MT of 0.5 and a VL of 0.25 work generally well. Apart from evaluating the effect of these values on the registration quality using TRE and NCC, we also studied the sensitivity (% true fiducials identified out of true number of fiducials) and precision (% true fiducials identified out of total number of fiducials identified) of the algorithm in fiducial detection. The above mentioned MT and VL values resulted in sensitivities of 60–100% and a precision range of 63–100% for fiducial detection for the Alexa Fluor 488/647 dual-colored tubulin samples (Additional file 1: Fig. S1A and B), indicating that the algorithm can detect the majority of the fiducial markers with a limited number of false positives. As illustrated in Additional file 1: Fig. S1C, the software has also the ability to remove the signal corresponding to the fiducial markers from the final image to yield a “cleaner” presentation.

To identify the fiducial markers from the STORM data the full frame set is processed by the algorithm by default. However, since we observed that there is some variation in the stability of the fiducial markers over the STORM acquisition (Additional file 1: Fig. S2), we wanted to test if reliable fiducial marker identification could be achieved with a smaller subset of acquired frames. We therefore investigated the effect of adjusting the number of frames analyzed on the identification of the fiducial markers and registration quality. In the case of Alexa Fluor 488/647 dual-colored tubulin samples we observed that the TRE decreased with an increasing number of analysed frames starting from frame 1 up until approximately 5000 frames for the 488 nm channel, and then slightly increased again at around 20,000 frames (Additional file 1: Fig. S3A). The included frame range of the 642 nm channel did not affect the TRE score for this combination. In the case of Alexa Fluor 568/647 dual-colored tubulin samples the TRE decreased until approximately 5000 frames for the 642 nm channel and remained stable after that, whereas the frame range of the 561 nm channel had no effect on the TRE (Additional file 1: Fig. S3B). These results suggest that it is possible to achieve high-quality registration even with a smaller subset of frames included in the fiducial marker identification, which has the benefit of reducing the processing time. However, since different types of fiducial markers behave differently in the STORM acquisition conditions and there is also variation between the channels, optimal frame range for RegiSTORM thus needs to be determined on a case-by-case basis.

### Limitations

In this work we used two metrics to evaluate the performance of the algorithm (i.e. the registration quality): TRE and NCC. Due to user-dependency and humane inaccuracies the manual annotation is somewhat error-prone, which is why the absolute TRE values calculated here likely have some inaccuracy. However, we were still able to see a significant improvement in this metric, and our values (around 40–60 nm) are approaching the resolution limit of STORM microscopy (around 20 nm [1]). In terms of NCC, a key limitation of this metric is that due to the staining approach and different blinking behaviour and intensities of different fluorophores in the STORM acquisition conditions, it is not possible to reach perfect pixel-wise correlation even in the case of successful registration. Therefore the calculated cross-correlation coefficients did not reach values close to 1 despite good quality registration.

To achieve high-quality registration using fiducial markers, the density and distribution of the fiducials in the sample, as well as inherent properties of the fiducial markers such as brightness and stability, play critical roles. Too low total count and a concentrated distribution of the fiducials, as well as positions in different focus levels when compared to the sample signal, can limit the algorithm's ability to identify them and calculate an accurate transformation matrix, particularly when it comes to the rotational component. All this can negatively impact the registration quality. On the other hand, if the fiducial density is too high or the fiducials are much brighter than the sample signal, there is a risk of masking the signal coming from the targets of interest, which can result in poor overall image quality. Although extensive evaluation and optimization of fiducial markers is beyond the scope of this work, these factors are good to keep in mind as potential error sources when conducting STORM channel registration utilizing fiducial markers.

## Conclusions

In this work we have developed a registration algorithm for sequentially acquired multi-color STORM images. The algorithm is capable of directly processing fluorophore localization data, as opposed to generated images, making it ideal for STORM. Its performance was initially demonstrated using only fiducial markers, illustrating that the algorithm can successfully identify the fiducials and apply a correcting transformation. Subsequently, the algorithm was shown to be functional in a more complex and experimentally relevant setup of multi-labeled microtubuli, suggesting the applicability of this tool in a wide range of experimental conditions in sequential multi-channel STORM imaging. Lastly, using the cluster mode of the algorithm for alignment, two-color STORM images of fluorescent cargo-loaded fluorescent LNPs were successfully registered in the absence of fiducial markers as references, which adds to the versatility of this tool. The algorithm is implemented in an open source software tool, RegiSTORM, providing a user-friendly GUI for easy selection of files, tuning parameters, conduction of the registration and saving the results. The software has been compiled into a Windows application so that users without experience in Python programming can easily use it. This tool has the potential to be instrumental in applying super-resolution microscopy to elucidate and accurately characterize a plethora of unknown nanoscale interactions (e.g. protein–protein, protein-organelle) in cell biology and nanomedicine, such as extracellular vesicle composition and signaling, endosomal escape pathways, signaling pathways in general and localization of carrier and active molecules in drug delivery systems [37–42]. The interpretation of such nanoscale interactions will be heavily biased in the absence of proper and accurate alignment of the channels representing the interacting targets.

## Methods

### Sample preparation for STORM imaging

To develop and test the RegiSTORM software three sample types were prepared and imaged using STORM: fiducial markers only, cells immunostained for tubulin with fiducial markers and fluorescent lipid nanoparticles (LNPs; composition depicted in Additional file 1: Table S3) loaded with a fluorescent cargo but no fiducial markers. For LNP size and fluorescence characterization see Additional file 1: Fig. S4. TetraSpeck™ microspheres (100 nm in diameter; Thermo Fisher Scientific, Waltham, MA, USA) were introduced to the samples right before the STORM imaging. Details of the sample preparation can be found in Additional file 1.

### STORM imaging and reconstruction

#### Image acquisition

Prior to imaging, the sample was soaked in imaging buffer with the following composition: Tris buffer (160 mM Tris, 40 mM NaCl, pH adjusted to 8.0), 10 wt% glucose, 0.5 mg/mL glucose oxidase from *Aspergillus niger* (G7141), 47 µg/mL catalase from bovine liver (C1345) and 10 mM cysteamine (pH adjusted to 8.0). All the components of the buffer were purchased from Sigma Aldrich (Saint Louis, MO, US). The μ-plate was further sealed with parafilm to decrease oxygen entry.

STORM imaging was conducted with a Nikon Ti Eclipse inverted microscope (Nikon, Tokyo Japan), with cube filters (excitation: Chroma ZET405/488/561/640x, emission: Chroma ZET405/488/561/640m) and TIRF dichroic ZET405/488/561/640bs, and equipped with Cairn laser module (Cairn Research, Kent, UK) with 200 mW 488 nm, 150 mW 561 nm and 140 mW 642 nm lasers. A CFI SR Apo TIRF 100X oil objective (N.A. 1.49) was used with a 1.5X Optovar lense, thus giving a final magnification of 150X. The camera (Andor iXON Ultra 888 EMCCD, Oxford Instruments, Belfast, UK) had a pixel size of 13 µm. The image acquisition was controlled with MetaMorph^®^ (Molecular Devices, San Jose, CA, US) and Micro-Manager open-source software [43]. The region of interest was set to 256 × 256 pixels. A diffraction-limited image was taken from each ROI for reference before starting the STORM acquisition. Around 30 000 frames, with an exposure time of 30 ms/frame, 100% laser power and electron multiplying gain of either 100 (642 & 561 lasers) or 300 (488 laser) were recorded for each image. Thus, each acquisition took around 15 min and was started once the photoswitching of the fluorophores was at an optimal level as visually evaluated by the user. In multicolour STORM imaging of the fiducial markers only and tubulin-stained cells, the channels were recorded sequentially, moving on from higher to lower excitation wavelength to minimize the damage caused to the fluorophores in the other channels. However, in case of the LNPs, the acquisition was conducted in the opposite order (first 488 nm channel and then 642 nm channel) due to the lower signal intensity from the Alexa Fluor 488 labeled LNP lipids when compared to the Alexa Fluor 647 labeled cargo. The recordings were stored in uncompressed TIFF format with a bit depth of 16 bits per channel.

#### Image reconstruction

The localization events and images from each channel were reconstructed with the ThunderSTORM plugin [44] in Fiji [15, 16], followed by drift correction using the plugin’s cross-correlation algorithm (see Supplementary information for reconstruction parameters). As a result, comma-separated values (csv) files with estimated x,y coordinates for all the detected localizations were obtained. In the case of the LNP sample in the 642 nm channel, localizations with intensity lower than 2000 were omitted to remove background and noise for improved registration. Prior to registration, chromatic aberration was corrected using the Detection of Molecules (DoM) plugin [4] in Fiji, using a correction transformation estimated from diffraction-limited images of densely arranged fiducial markers only. The output .csv files were used as the input for RegiSTORM. RegiSTORM saves the results in new.csv files, which can be re-imported to Fiji or processed using other software for visualization and further analysis. To visualize the data, the Normalized Gaussian method in ThunderSTORM was used, with a magnification of 10 and an uncertainty value calculated on a per-image basis.

### Manual annotation

Manual annotation of the fiducial markers in both the fiducials only and tubulin STORM images was conducted with the ImageJ annotation tool by a single expert (M.O.). Briefly, by using the in-built multi-point tool the corresponding fiducials were clicked in all the images aiming to hit the very center of each dot. Thereafter the x,y coordinates of these selections were saved as a .txt file. Unclear targets or fiducial markers not observed in all the channels were omitted. In case of two-color tubulin samples the third channel (with signal only from the fiducial markers) was used to guide in the annotation process. Manual annotation with the same approach was also conducted for the two-color LNP STORM images by roughly estimating the middle point of the LNPs. Non-loaded LNPs and signals from free cargo were omitted from the annotation. To improve the accuracy of the manual annotation, for each annotated point, we identified the localization coordinates in the STORM dataset closest to the manually annotated coordinates.

### Multi-channel registration

RegiSTORM works by first identifying the fiducial markers, based on their non-blinking behaviour, in a reference channel and in one or more moving channel(s). After identifying the fiducials, it performs a rigid transformation to match the fiducial coordinates of the moving channel(s) to those of the reference channel. A detailed description of the registration algorithm is provided in the following subsections.

#### Fiducial detection

In the first part of the algorithm the fiducial markers in each channel are identified as follows:

1. Import STORM data (.csv format) reconstructed from the original image frames with a reconstruction software (e.g. ThunderSTORM [44]).
2. Identify the frame with the highest number of localizations. All these localizations are initially considered as potential fiducials.
3. Go through the remaining frames and search for localizations that are within a tolerance *r* equaling the fiducial diameter (100 nm) from the coordinates of each potential fiducial. This is conducted using a KD-tree for quick nearest-neighbor lookup [45].
4. For each candidate fiducial *c,* apply two inclusion criteria, mean tolerance (MT, Eq. 1) and variance limit (VL, Eq. 2) calculated over all frames *f* = 1, …, *n*, to refine the list of identified fiducials.
  1 $$MT = \frac{1}{n}\mathop \sum \limits_{f = 1}^{n} \left| {N_{f} } \right|$$
  2 $$VL = \frac{1}{n}\mathop \sum \limits_{f = 1}^{n} \left( {\left| {N_{f} } \right| - MT} \right)^{2}$$
  where *N*_*f*_ (Eq. 3) is the set of localizations *x*_*f*_ in frame *f* within an Euclidean distance *d* smaller than the tolerance *r* from the candidate fiducial *c:*
  3 $$N_{f} : = \left\{ {x_{f} \in X_{f} \left| {d\left( {x_{f} ,c} \right) < \left. r \right\} } \right.} \right.$$
  Unless otherwise specified we used a minimum acceptable MT of 0.5 and a maximum acceptable VL of 0.25 in our analyses, selected based on a parameter grid search (see Fig. 5). Only candidate fiducials fulfilling these two criteria are included. This retains the emitters which are in a stable emitting state over time.
5. Transferring the coordinates of the identified fiducials to the transformation section.

RegiSTORM additionally allows the user to constrain the fiducial detection to rely on a subset of the captured frames in order to exclude frames with decreased fluorescence emission. In our analyses only the 10 000 first frames of the 488 nm channel were used for this step, since very little signal was detected from the fiducials in this channel in the remaining frames. In the 642 nm and 561 nm channels the fiducial markers were more stable for the full length of the acquisition. The stability of the TetraSpeck™ fiducial markers in different channels over the course of the image acquisition is illustrated in Additional file 1: Fig. S2.

#### Estimation of transformations

After identifying the fiducial markers the registration is performed according to the following steps:

1. Define the reference channel and the moving channel(s), i.e. the channels being transformed.
2. Apply the Iterative Closest Point (ICP) algorithm [33, 34] to each of the moving channels to align the sets of fiducials with those of the reference channel. In the ICP algorithm, a filter is applied when finding the nearest-neighbor for each point across the channels. By considering all the distances to the nearest-neighbors between the two sets, the mean distance and standard deviation is calculated. Point pairs with distance more than one standard deviation larger than the mean distance are considered outliers. This process was repeated until convergence, which was determined by the change in mean distance being less than 1 nm between two consecutive iterations.
3. Apply the best-fit rigid transformations (final transformations from the iteration step above) estimated for the identified fiducials to all the localizations in the channels being corrected. A rigid transformation has a rotational and a translational component [32].
4. Output the corrected localization lists as .csv-files.

#### Removing fiducials

RegiSTORM has also an optional feature to remove the fiducial markers from the dataset for fiducial-free visualization and image analysis. This is accomplished by removing all the localizations within a distance of one fiducial diameter around the coordinates of the identified fiducials. The performance of this functionality is illustrated for Alexa Fluor 488/647 dual-colored tubulin samples in Additional file 1: Fig. S1.

#### Cluster mode

In addition to the fiducial marker based image registration RegiSTORM has an alternative mode where clusters of signals are identified instead of fiducials. In the cluster mode the algorithm works similarly to the fiducial marker identification, with the exception that the MT and VL criteria are not applied. Instead, for each localization representing a potential cluster centre, the number of neighbouring localizations within an Euclidean distance *r*_*c*_ specified by the user is computed across all frames. The 25 localizations with the largest number of neighbours are retained as the identified clusters and used as landmarks for registration. The rigid alignment matrix transformation step remains the same. To demonstrate its feasibility we have used the cluster mode to register two-color STORM images of fluorescent cargo loaded labeled LNPs (Fig. 4). LNPs and the cargo therefore serve as “landmark clusters” with high expectancy of co-localization. A distance *r*_*c*_ of 750 nm was used for these images.

### Quantification of results and statistical analysis

Two metrics were used for the quantification of image registration accuracy: (1) Target Registration Error (TRE, Eq. 4) which measures the deviation of landmark points from their expected locations in terms of Euclidean distance in the detection channels. We calculated TRE for the manually annotated fiducials/LNPs between the different channels before and after applying registration to the localization data; (2) NCC analysis (Eq. 5), which measures pixel-wise similarity of two images, one per channel, in terms of correlation between the intensities of corresponding pixels [35]. We calculated NCC for images reconstructed from the tubulin and LNP datasets.

4 $$\begin{array}{*{20}c} {TRE = X_{i} - X_{j} } \\ \end{array}$$

5 $$\begin{array}{*{20}c} {NCC = \frac{{\mathop \sum \nolimits_{i = 0}^{N} \mathop \sum \nolimits_{j = 0}^{M} \left[ {\left( {I_{1, ji} - I_{1, m} } \right)*\left( {I_{2, ij} - I_{2, m} } \right)} \right]}}{{N*M*\sigma_{1} *\sigma_{2} }} } \\ \end{array}$$

For the TRE X_i_ is the coordinate of the fiducial in the moving channel and X_j_ is the corresponding fiducial in the reference channel. For the NCC, N and M is the number of pixels in each dimension. I is the intensity at pixel i,j where $i \in \left\{1, \dots , N\right\}, j \in \left\{1, \dots , M\right\}$, and 1 and 2 refers to the moving and reference channel respectively. I_m_ refers to the mean intensity and σ is the standard deviation of the intensity for the given image.

A two-sided Wilcoxon signed rank test was used to evaluate statistical significance of differences in TRE before and after registration. A significance level of 0.05 was used. In the box plot visualizations of the quantitative data the box shows the data from lower to upper quartile, with median marked with a line, whereas the whiskers indicate the minimum and maximum. Automatically identified outliers are not shown in the plots. The number of fiducial markers/LNPs used for the TRE calculations is provided in Additional file 1: Table S4. Additional file 1: Table S5 shows the *p* values for the TRE calculations in the different datasets.

### Software implementation

RegiSTORM was developed using Python 3.8 and can be used either in a Python interpreter or as a Windows executable application. Dependencies include: Joblib, SciPy, SkLearn, OS, Pandas and Numpy. The GUI was implemented using the PySimpleGUI library, and the software was compiled into a Windows executable file using PyInstaller. The build was then compiled into an installable file using Inno Setup (JrSoftware) allowing the installation of the software on Windows computers.

In the GUI, the user can build up a list of jobs, where each job is one set of registrations. When the user has defined all the required jobs and clicks ‘run’, the software will sequentially apply the algorithm to all the jobs in the task list (see documentation for the Github file).

## Supplementary Information

**Additional file** 1. Supplementary methods, supplementary figures S1-S4, supplementary tables S1-S5. 

## Data Availability

The datasets generated and analysed during the current study are available in Zenodo repository (10.5281/zenodo.5509861). RegiSTORM is also freely available as open-source (MIT license) at https://github.com/oystein676/RegiSTORM.git.

## Abbreviations

AF: Alexa Fluor
GUI: Graphical user interface
hBMSC: Human bone marrow-derived stromal cells
ICP: Iterative closest point
LNP: Lipid nanoparticle
MT: Mean tolerance
NCC: Normalized cross-correlation
SD: Standard deviation
STORM: Stochastic optical reconstruction microscopy
TRE: Target registration error
VL: Variance limit
