# Supplementary material for: RegiSTORM: channel registration for multi-color stochastic optical reconstruction microscopy
Source: BMC Bioinformatics. 2023 Jun 5;24:237. doi: 10.1186/s12859-023-05320-1 (PMC10242778; doi:10.1186/s12859-023-05320-1)
Supplement: Supplementary file 1 — Additional file 1. Supplementary methods, supplementary figures S1-S4, supplementary tables S1-S5. [file 12859_2023_5320_MOESM1_ESM.docx]

**Additional file 1**

**RegiSTORM: channel registration tool for multi-color stochastic optical reconstruction microscopy**

Øystein Øvrebø^1*^, Miina Ojansivu^2*^, Kimmo Kartasalo^3^, Hanna M. G. Barriga^2^, Petter Ranefall^4^, Margaret N. Holme^2^, Molly M. Stevens^1,2^

1 Department of Materials, Department of Bioengineering and Institute of Biomedical Engineering, Imperial College London, London, UK

2 Department of Medical Biochemistry and Biophysics, Karolinska Institute, Stockholm, Sweden

3 Department of Medical Epidemiology and Biostatistics, Karolinska Institute, Stockholm, Sweden

4 SciLifeLab BioImage Informatics Facility, and Department of Information Technology, Uppsala University, Uppsala, Sweden

*equal contribution

**
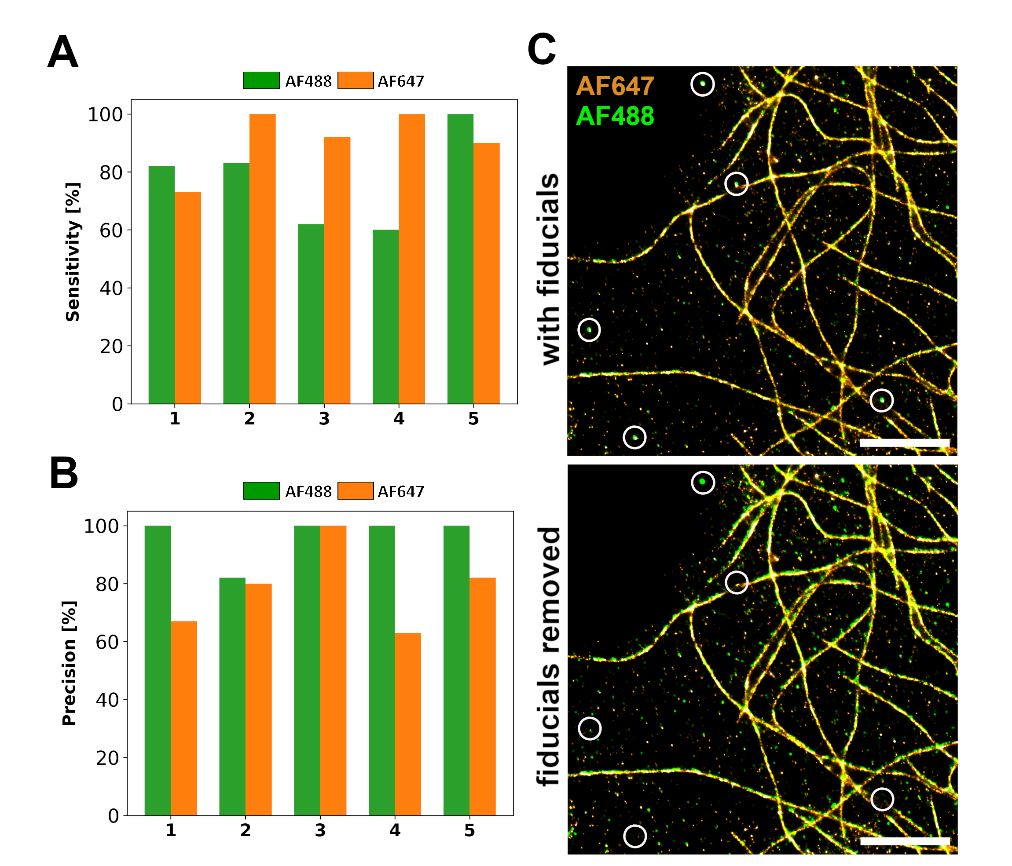
**

**Supplementary Figure 1.** Evaluation of the fiducial marker removal function of the RegiSTORM software. **A.** Sensitivity-% of the algorithm’s fiducial marker detection for 5 STORM images of Alexa Fluor (AF) 488/647 dual-labelled tubulin. **B.** Precision-% of the algorithm’s fiducial marker detection for 5 STORM images of AF488/AF647 dual-labelled tubulin. **C.** Representative STORM images of dual-labelled tubulin before and after the removal of fiducial markers, imaged with 488 and 642 nm excitation. The areas containing fiducial markers are indicated with white circles. Scale bars = 3 μm.

**
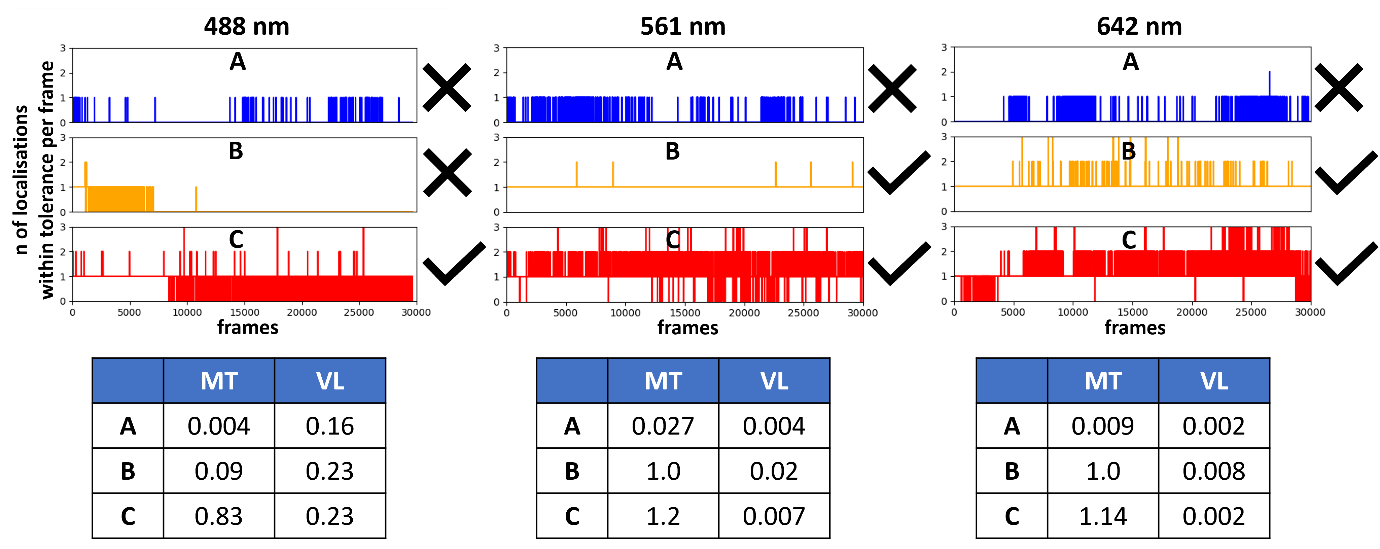
**

**Supplementary Figure 2.** Evaluation of the TetraSpeck^TM^ fiducial emission over the course of the image acquisition. Three example fiducial candidates (A-C) are depicted for each channel. The candidates qualified as fiducial markers by the algorithm are marked with ✔, whereas the rejected candidates are marked with **X**. Mean tolerance (MT) and variance limit (VL) are presented for all the fiducial candidates in the graphs. A MT of > 0.5 and VL of < 0.25 were used as detection criteria.

**
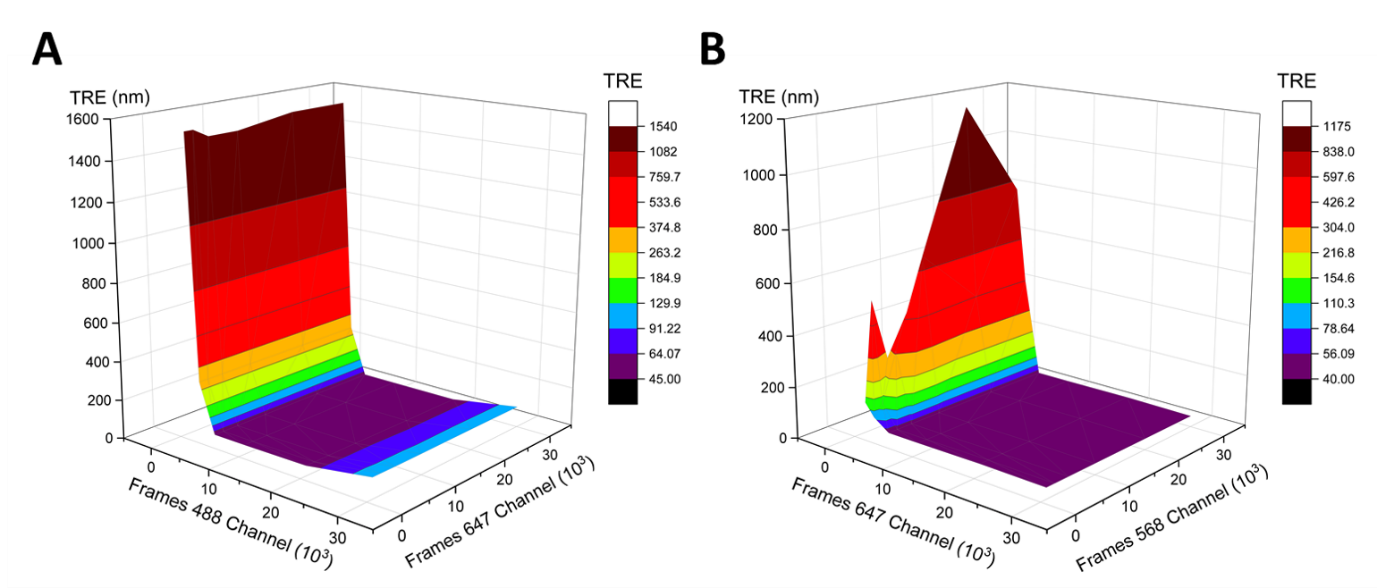
**

**Supplementary Figure 3.** The effect of the number of included frames on the algorithm’s performance. **A.** Target Registration Error (TRE) analysis conducted with varying frame numbers of Alexa Fluor 488/647 dual-labelled tubulin STORM images. **B.** TRE analysis conducted with varying frame numbers of Alexa Fluor 568/647 dual-labelled tubulin STORM images.

**Sample preparation**

***Fiducial markers only***

TetraSpeck^TM^ microspheres (100 nm in diameter; Thermo Fisher Scientific, Waltham, MA, USA) were used as fiducial markers in all the experiments. To image the fiducial markers alone, they were diluted 1:50 v/v in DPBS buffer (Gibco, Thermo Fisher Scientific) and incubated in the wells of 8-well glass bottom μ-slides (Ibidi, Gräfelfing, Germany) for 20 min at room temperature, followed by one DPBS wash. Samples were always imaged fresh.

***Cell culture***

Primary human bone marrow-derived stromal cells (hBMSCs) were purchased from RoosterBio (Frederick, Maryland, USA) and cultured in medium containing 10% v/v fetal bovine serum (FBS; Sigma Aldrich, Saint Louis, MO, USA) and 1% v/v Penicillin/Streptomycin (10 000 U/mL; Gibco, Thermo Fisher Scientific) in Minimum Essential Medium alpha (Gibco, Thermo Fisher Scientific). For STORM imaging, hBMSCs were plated on 8-well glass bottom μ-slides (Ibidi) at a density of 15 000 cells/cm^2^ and allowed to attach overnight. Human BMSCs of passage 5 were used.

***Immunocytochemical staining***

Prior to imaging, cells were fixed by washing samples once with DPBS buffer (Thermo Fisher Scientific) at 37 ºC and then incubating for 10 min at room temperature in 4% v/v paraformaldehyde solution (PFA; prepared from 20% v/v solution (Electron Microscopy Sciences, Hatfield, PA, USA) by diluting in DPBS) containing 0.2% v/v glutaraldehyde (Merck, Darmstadt, Germany). After washes the samples were stored in DPBS at 4 ºC until the staining and imaging (max. two months). To quench autofluorescence the samples were treated with 0.1% w/v sodium borohydride (Sigma Aldrich; in DPBS) for 10 min at room temperature, followed by extensive washing with DPBS. Samples were permeabilized with 0.2% v/v Triton X-100 (Sigma Aldrich) in DPBS for 5 min followed by blocking in 3% w/v bovine serum albumin (BSA; Thermo Fisher Scientific) solution for 1.5 hours at room temperature. Primary antibody (mouse monoclonal anti-tubulin; T5168, Sigma Aldrich) was diluted 1:2000 v/v in 3% w/v BSA and incubated for 1.5 hours at room temperature, followed by extensive DPBS washes. The following secondary antibodies were used: Alexa Fluor 647 goat anti-mouse IgG (A21235, Invitrogen, Thermo Fisher Scientific), Alexa Fluor 568 goat anti-mouse IgG (A11004, Invitrogen, Thermo Fisher Scientific), Alexa Fluor 488 Plus goat anti-mouse IgG (A32723, Invitrogen, Thermo Fisher Scientific). All were diluted 1:1000 v/v in 3% w/v BSA and incubated for 1.5 hours at room temperature. To after fixate the antibodies, samples were treated with 2% v/v PFA (prepared from 20% v/v solution (Electron Microscopy Sciences) by diluting in DPBS) for 10 min at room temperature. The samples were stored in DPBS at +4 ºC until imaged (max. two days). Immediately prior to imaging TetraSpeck^TM^ fiducial markers were introduced to the samples as described in section 2.1.1.

***Lipid film preparation, LNP formulation, loading and immobilization***

Lipids used: 1-Oleoyl-*rac*-glycerol (monoolein, MO; M7765, Sigma Aldrich), cholesterol (CHOL; 700000P, Sigma Aldrich), 1,2-dioleoyl-*sn*-glycero-3-phosphate (sodium salt) (DOPA; 840875P, Sigma Aldrich), biotin *N*-(biotinoyl)-1,2-dihexadecanoyl-*sn*-glycero-3-phospho- ethanolamine (triethylammonium salt) (biotin-DHPE; B1550, Thermo Fisher Scientific), 1,2-dioleoyl-sn-glycero-3- phosphoethanolamine-N-(TopFluor® AF488) (ammonium salt) (PE-AF488; 810386C, Sigma Adrich).

Lipid films of the composition indicated in **Supplementary Table 3** were prepared by mixing from stock lipid chloroform solutions according to the ratios shown in **Supplementary Table 3**, followed by evaporation of the solvent in a fume hood overnight and freeze-drying for 4 hours to remove the residual chloroform. Films were stored sealed in a nitrogen atmosphere at -20 °C until LNP formulation. To formulate LNPs, lipid films were hydrated in DPBS containing Mg^2+^ and Ca^2+^ (Thermo Fisher Scientific), followed by incubation on a +60 ºC hot plate for 30 min. Pluronic F127 stabilizer (Sigma Aldrich) was added at a ratio of 2.5 mol% vs lipid content to give a final lipid concentration of 5 mM. The samples were tip-sonicated (Vibra-Cell^TM^, Sonics & Materials, Newtown, CT, USA) using pulse mode (1 s on, 1 s off, 30% maximum amplitude for 3 x 5 min), followed by 30 s of vortexing. Residual titanium from the sonication tip was removed via centrifugation (4000 rpm for 10 min in a benchtop centrifuge). LNPs were characterised by dynamic light scattering (DLS; Zetasizer Nano ZS, Malvern, UK) which showed a single peak at around 150 nm with polydispersity index (PDI) < 0.2 (**Supplementary Figure 4A**). LNPs were loaded with Streptavidin by mixing the LNP preparation (179 µL, 5 mM) with Streptavidin Alexa Fluor 647 (25 µL, 2 mg/mL) (Invitrogen, Thermo Fisher Scientific) and DPBS (296 µL). Unencapsulated Streptavidin was removed by size exclusion chromatography on Sepharose^®^ CL-2B (Sigma Aldrich). Five x 1 mL and 9 x 2 mL fractions were collected and the fluorescence of the fractions was measured with a Varioscan Lux plate reader (Thermo Fisher Scientific) at 494/520 and 650/680 nm (ex./em.) (**Supplementary Figure 4B**). Loaded LNPs were found in fraction 7, which was also verified with DLS (**Supplementary Figure 4A**). As expected, protein association with the LNPs increased the size of the loaded LNPs when compared to non-loaded ones, and slightly increased their polydispersity; however, both loaded and non-loaded LNPs formed a rather homogeneous solution (polydispersity indices ≤ 0.2). LNPs were immobilised for imaging via charge interactions. The 8-well glass bottom μ-slides (Ibidi) were rendered positively charged by treating with 0.1% v/v poly(ethyleneimine) solution (Sigma Aldrich) for 20 min at room temperature. The undiluted (negatively charged) LNPs were then incubated on the wells for one hour at room temperature and imaged immediately.


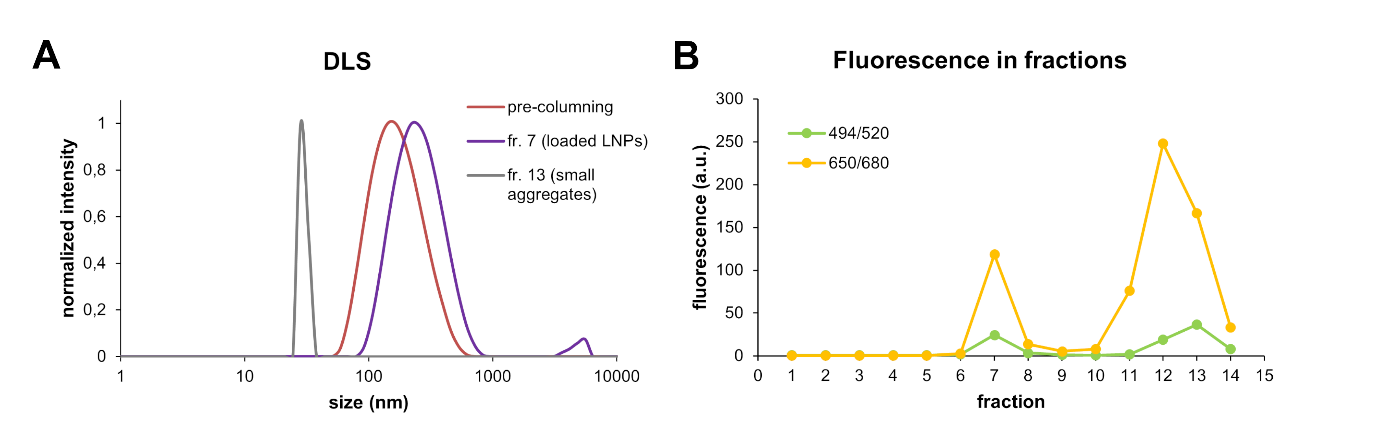


**Supplementary Figure 4.** LNP characterisation. **A.** Dynamic light scattering (DLS) of non-loaded LNPs and loaded LNPs from size exclusion column fractions 7 and 13. Based on these data LNPs were in fraction 7 whereas fractions 11-13 contain free protein. The samples had the following Z-Averages and polydispersity indices (PDIs): non-loaded 153 nm (PDI = 0.158); fraction 7, 237 nm (PDI = 0.208); fraction 13, 5994 nm (PDI = 1). **B.** Fluorescence of the fractions from size-exclusion chromatography as measured by plate reader at excitation/emission 494/520 and 650/680 nm.

**STORM image reconstruction parameters used in ThunderSTORM**

Image Filtering

Filter: Difference-of-Gaussians filter (Sigma1 = 1.0 px, Sigma2 = 1.6 px)

Approximate localization of molecules

Method: Local maximum

Peak intensity threshold: std(Wave.F1)

Connectivity: 8-neighbourhood

Sub-pixel localization of molecules

Method: PSF: Integrated Gaussian

Fitting radius (px): 3

Fitting method: Weighted least squares

Initial sigma (px): 1.6

Multi-emitter fitting analysis: enabled

Maximum of molecules per fitting region: 3

Model selection threshold (p-value): 1.0E-6

The intensity range (photons) is not limited.

[1]

**Supplementary Table 1.**

**
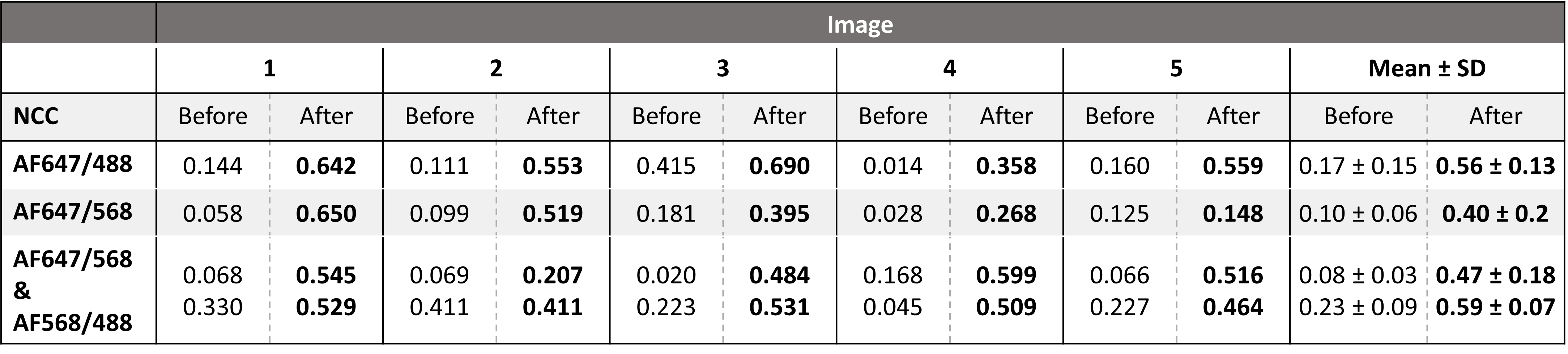
**NCC values for the multicolor tubulin STORM images. NCC = normalized cross-correlation.

**Supplementary Table 2.**

**
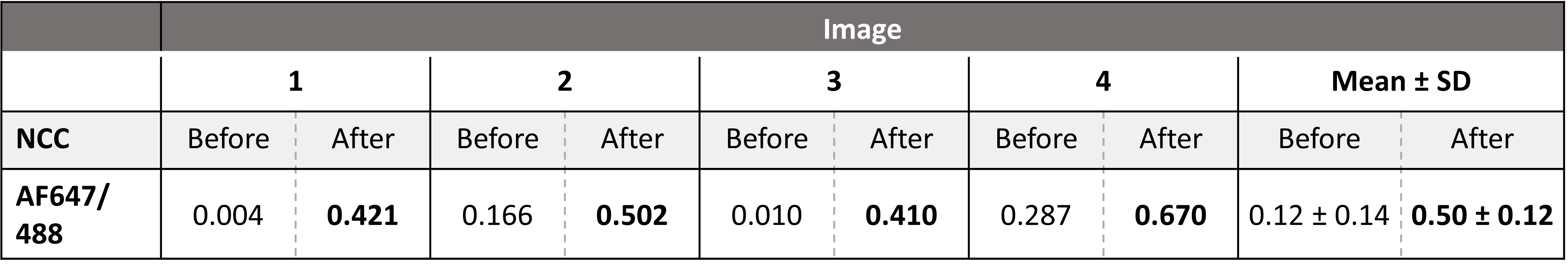
**

NCC values for the dual-color LNP STORM images. NCC = normalized cross-correlation.

**Supplementary Table 3.**

| **Lipid** | **mol%** | **Solvent** |
| --- | --- | --- |
| MO | 60 | chloroform |
| CHOL | 30 | chloroform |
| DOPA | 10 | chloroform |
| biotin-DHPE | 0.1 | chloroform |
| PE-AF488 | 0.1 | methanol |

Lipid composition of the LNPs. Biotin-DHPE and PE-AF488 were added to the MO-CHOL-DOPA lipid films afterwards at 0.1 mol%. AF = Alexa Fluor.

**Supplementary Table 4.**

**
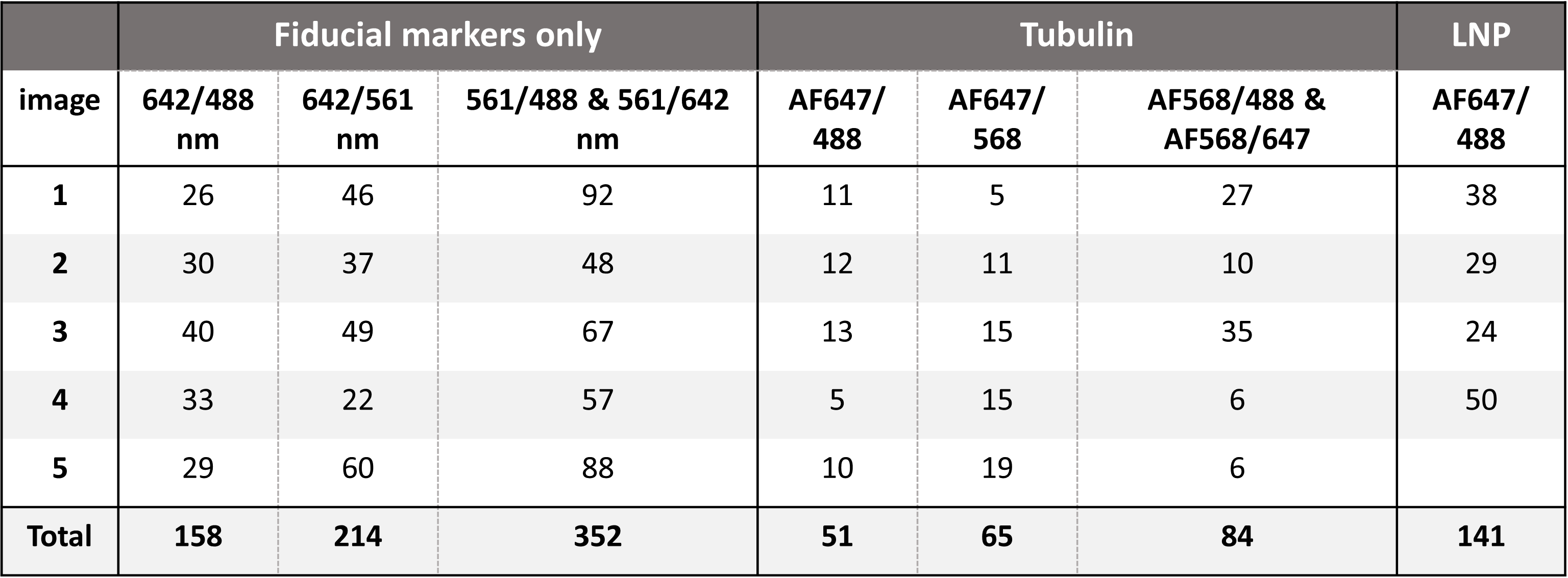
**

Number of fiducial markers/LNPs used for the TRE calculation in the different datasets. TRE = target registration error, LNP = lipid nanoparticle, AF = Alexa Fluor.

**Supplementary Table 5.**

**
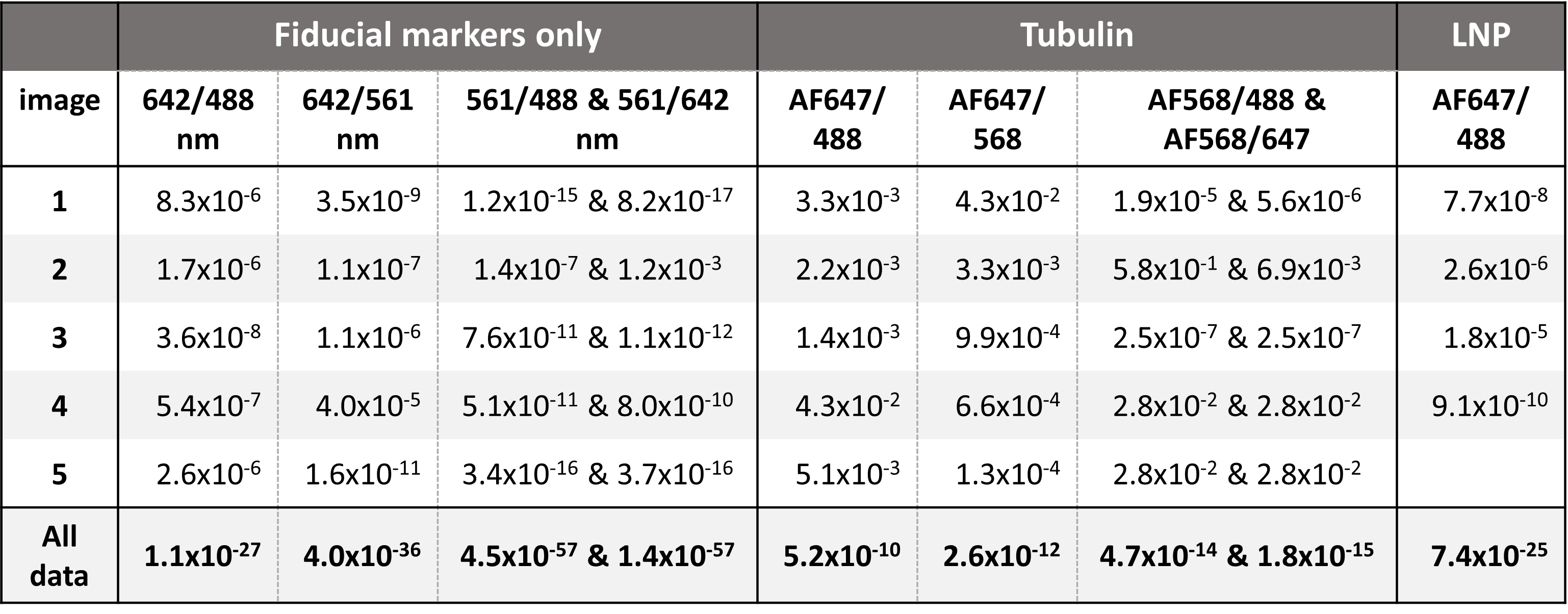
**

p-values for the TRE in the different datasets. TRE = target registration error, LNP = lipid nanoparticle, AF = Alexa Fluor.

**References**

[1] M. Ovesný, P. Křížek, J. Borkovec, Z. Švindrych, G.M. Hagen, ThunderSTORM: a comprehensive ImageJ plug-in for PALM and STORM data analysis and super-resolution imaging, Bioinformatics. 30 (2014) 2389–2390.
